# Crystal structure of chlorido­[*trans*-1-(di­phenyl­phosphane­thioyl-κ*S*)-2-(di­phenyl­phosphano­yl)ethene]gold(I) di­chloro­methane hemisolvate^1^

**DOI:** 10.1107/S2056989016009816

**Published:** 2016-06-21

**Authors:** Christina Taouss, Peter G. Jones

**Affiliations:** a Institut für Anorganische und Analytische Chemie, Technische Universität Braunschweig, Postfach 3329, D-38023 Braunschweig, Germany

**Keywords:** crystal structure, gold, phosphine, chalcogenide

## Abstract

The title compound, [AuCl(C_26_H_22_OP_2_S)]·0.5CH_2_Cl_2_, crystallizes with a *trans*-O—P⋯P—S geometry of the groups either side of the C=C double bond, which prevents any intra­molecular contact between the Au and O atoms. The Au^I^ atom exhibits a nearly linear coordination [Cl—Au—S = 177.55 (4)°]. The mol­ecules associate to form broad ribbons parallel to the *c* axis *via* two C—H⋯O, one C—H⋯Cl(Au) and one Au⋯Cl inter­action.

## Abstract 2

The title compound crystallizes with a *trans*-O—P⋯P—S geometry of the groups either side of the C=C double bond.

## Chemical context   

We are inter­ested in phosphine chalcogenide complexes of gold (Taouss & Jones, 2016 ▸, and references therein). In general, we have synthesized complexes *L*Au*X*, where *L* is a phosphine chalcogenide and *X* is chlorine or bromine, and then oxidized these first to gold(III) complexes *L*Au*X*_3_ and further to (*LX*)^+^(Au*X*_4_)^−^. The title compound was obtained as an unexpected *trans* product in minimal yield (a few small crystals) during attempts to recrystallize *cis*-(Ph_2_PC=CPPh_2_S)AuCl (Taouss & Jones, 2014 ▸). The oxidation of the second P atom to P=O, presumably by atmospheric oxygen, is not unusual, but we are at a loss to explain the change of configuration at the C=C bond from *cis* to *trans*. One possibility, in view of the small amounts involved, is that the *cis* diphosphine as purchased contained a small amount of *trans* impurity.
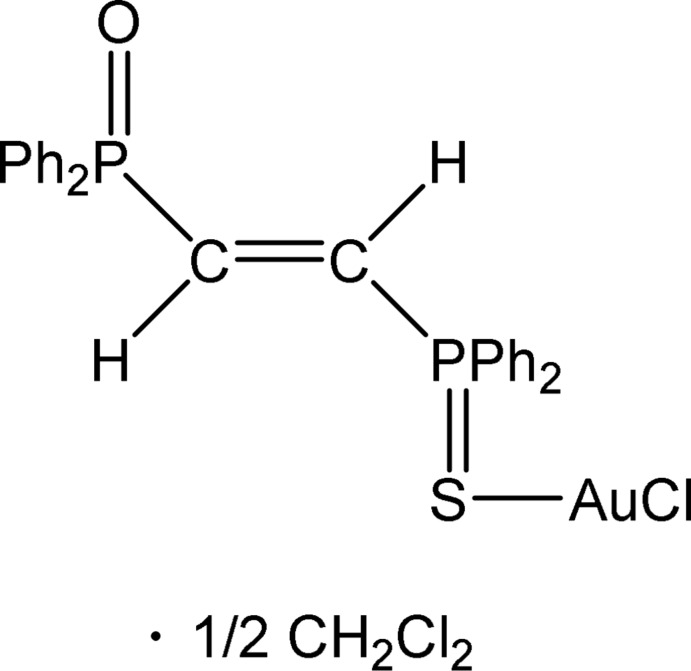


## Structural commentary   

**Figure 1:**
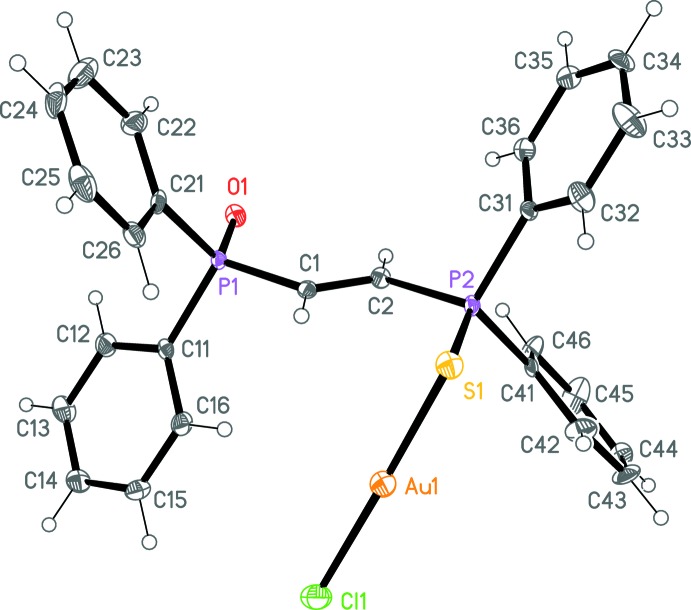
The mol­ecule of the title compound in the crystal. Ellipsoids correspond to 50% probability levels. The disordered solvent is not shown.

The molecular structure of the title compound is shown in Fig. 1 ▸. In the absence of a free phospho­rus donor atom, the gold(I) atom is, as expected, coordinated by the softer sulfur donor rather than the oxygen. Bond lengths and angles are essentially as expected (Table 1 ▸). The P=S bond is somewhat lengthened compared to non-coordinating phosphine sulfides (see Section 4). The torsion angle O1—P1⋯P2—S1 is 174.72 (12)°, which is similar to the values observed for dppe-derived complexes of the type *E*=PPh_2_CH_2_CH_2_PPh_2_Au*X* (*E* = chalcogen and *X* = halogen); the dppm analogues *E*=PPh_2_CH_2_PPh_2_Au*X*, however, tend to display corresponding torsion angles close to zero, thus promoting short intra­molecular Au⋯E contacts (Taouss & Jones, 2014 ▸). The Au⋯O distance in the title compound [6.127 (3) Å] is clearly far too long for any significant inter­action.

## Supra­molecular features   

**Figure 2:**
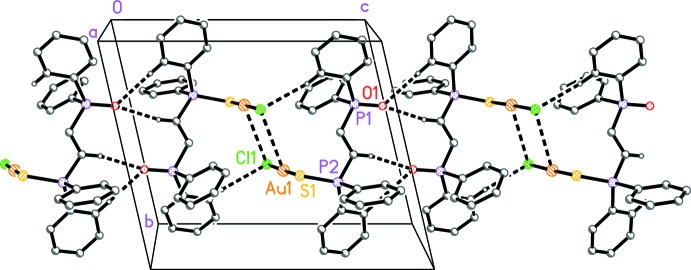
Packing diagram of the title compound viewed perpendicular to (100). ‘Weak’ C—H⋯O hydrogen bonds and Au⋯Cl contacts are drawn as thick dashed lines. Solvent molecules have been omitted for clarity.

The mol­ecules are connected into broad ribbons parallel to the *c* axis (Fig. 2 ▸) by the two shortest C—H⋯O and a C—H⋯Cl(Au) inter­action (Table 2 ▸), together with an Au1⋯Cl1 contact of 3.6522 (12) Å (symmetry code: −*x* + 1, −*y* + 1, −*z* + 1). The corresponding Au1⋯Au1 contact of 3.9827 (4) Å is probably less significant. The third C—H⋯O contact (not shown in Fig. 2 ▸) links the ribbons in the *a*-axis direction.

## Database survey   

A search of the Cambridge Structural Database (Groom & Allen, 2014 ▸; Groom *et al.*, 2016 ▸) (Version 5.37, 2015) revealed a mean P=S bond length of 1.954 Å for 485 examples of the non-coordinating moiety Ph_2_P(=S)C. This increases to 2.025 Å on coordination to an AuCl fragment (7 examples).

Perhaps surprisingly, there seem to be no structures of simple diphosphine dichalcogenides with the chalcogen atom(s) bonded to gold. One relevant publication, however, is that of the cyano-substituted derivative Ph_3_PAu[S=PPh_2_—C(CN)—PPh_2_=S] (Sithole *et al.*, 2016 ▸). This has a torsion angle of 70° across the atom sequence S=P⋯P=S because the formally noncoordinating S atom makes a short contact of 2.98 Å to the Au atom.

## Synthesis and crystallization   

Starting from *cis*-(di­phenyl­phosphan­yl)ethene, we generated the mono­sulfide and then the gold complex *cis*-(Ph_2_PC=CPPh_2_S)AuCl by reaction with (tetra­hydro­thio­phene)AuCl. This compound was successfully crystallized and its structure determined (Taouss & Jones, 2014 ▸). On one occasion, however, a few small crystals were obtained that proved not to be the intended compound, but instead the title compound.

## Refinement   

Crystal data, data collection and structure refinement details are summarized in Table 3 ▸. H atoms were included using a riding model starting from calculated positions, with C—H distances fixed at 0.95 Å. The di­chloro­methane mol­ecule is disordered over an inversion centre; appropriate restraints were employed to improve refinement stability, but the dimensions of disordered groups should be inter­preted with caution.

## Supplementary Material

Crystal structure: contains datablock(s) I, global. DOI: 10.1107/S2056989016009816/pj2031sup1.cif

Structure factors: contains datablock(s) I. DOI: 10.1107/S2056989016009816/pj2031Isup2.hkl

CCDC reference: 1486017

Additional supporting information: crystallographic information; 3D view; checkCIF report

## Figures and Tables

**Table 1:** Selected geometric parameters (Å, °)

Au1—Cl1	2.2726 (12)	P2—S1	2.0135 (16)
Au1—S1	2.2846 (11)	C1—C2	1.330 (6)
O1—P1	1.484 (3)		
			
Cl1—Au1—S1	177.55 (4)	C2—P2—S1	112.98 (15)
O1—P1—C1	114.90 (18)	P2—S1—Au1	100.06 (5)
			
P1—C1—C2—P2	176.8 (2)		

**Table 2:** Hydrogen-bond geometry (Å, °)

*D*—H⋯*A*	*D*—H	H⋯*A*	*D*⋯*A*	*D*—H⋯*A*
C2—H2⋯O1^i^	0.95	2.36	3.294 (5)	166
C46—H46⋯O1^i^	0.95	2.49	3.438 (5)	179
C26—H26⋯Cl1^ii^	0.95	2.75	3.583 (5)	147
C34—H34⋯O1^iii^	0.95	2.54	3.478 (5)	170

Symmetry codes: (i) 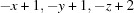; (ii) 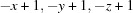; (iii) 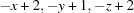.

**Table 3:** Experimental details

Crystal data	
Chemical formula	[AuCl(C_26_H_22_OP_2_S)]·0.5CH_2_Cl_2_
*M*_r_	719.32
Crystal system, space group	Triclinic, *P* 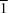
Temperature (K)	103
*a*, *b*, *c* (Å)	8.4458 (3), 11.4318 (5), 13.8713 (6)
α, β, γ (°)	76.940 (5), 85.785 (5), 77.541 (5)
*V* (Å^3^)	1273.49 (9)
*Z*	2
Radiation type	Mo *K*α
μ (mm^−1^)	6.21
Crystal size (mm)	0.16 × 0.16 × 0.05

Data collection	
Diffractometer	Oxford Diffraction Xcalibur Eos
Absorption correction	Multi-scan (*CrysAlis PRO*; Agilent, 2010 ▸)
*T*_min_, *T*_max_	0.644, 1.000
No. of measured, independent and observed [*I* > 2σ(*I*)] reflections	45210, 5824, 4835
*R*_int_	0.055
(sin θ/λ)_max_ (Å^−1^)	0.649

Refinement	
*R*[*F* ^2^ > 2σ(*F* ^2^)], *wR*(*F* ^2^), *S*	0.028, 0.070, 0.98
No. of reflections	5824
No. of parameters	311
No. of restraints	19
H-atom treatment	H-atom parameters constrained
Δρ_max_, Δρ_min_ (e Å^−3^)	1.83, −0.86

Computer programs: *CrysAlis PRO* (Agilent, 2010 ▸), *SHELXS97* and *SHELXL97* (Sheldrick, 2008 ▸) and *XP* (Siemens, 1994 ▸).

## supplementary crystallographic information

### Crystal data

**Table:**

[AuCl(C_26_H_22_OP_2_S)]·0.5CH_2_Cl_2_	*Z* = 2
*M**_r_* = 719.32	*F*(000) = 698
Triclinic, *P*1	*D*_x_ = 1.876 Mg m^−^^3^
*a* = 8.4458 (3) Å	Mo *K*α radiation, λ = 0.71073 Å
*b* = 11.4318 (5) Å	Cell parameters from 18482 reflections
*c* = 13.8713 (6) Å	θ = 2.1–30.7°
α = 76.940 (5)°	µ = 6.21 mm^−^^1^
β = 85.785 (5)°	*T* = 103 K
γ = 77.541 (5)°	Plate, pale yellow
*V* = 1273.49 (9) Å^3^	0.16 × 0.16 × 0.05 mm

### Data collection

**Table:**

Oxford Diffraction Xcalibur Eos diffractometer	5824 independent reflections
Radiation source: Enhance (Mo) X-ray Source	4835 reflections with *I* > 2σ(*I*)
Graphite monochromator	*R*_int_ = 0.055
Detector resolution: 16.1419 pixels mm^-1^	θ_max_ = 27.5°, θ_min_ = 2.1°
ω–scan	*h* = −10→10
Absorption correction: multi-scan (CrysAlis PRO; Agilent, 2010)	*k* = −14→14
*T*_min_ = 0.644, *T*_max_ = 1.000	*l* = −18→18
45210 measured reflections	

### Refinement

**Table:**

Refinement on *F*^2^	Primary atom site location: structure-invariant direct methods
Least-squares matrix: full	Secondary atom site location: difference Fourier map
*R*[*F*^2^ > 2σ(*F*^2^)] = 0.028	Hydrogen site location: inferred from neighbouring sites
*wR*(*F*^2^) = 0.070	H-atom parameters constrained
*S* = 0.98	*w* = 1/[σ^2^(*F*_o_^2^) + (0.039*P*)^2^] where *P* = (*F*_o_^2^ + 2*F*_c_^2^)/3
5824 reflections	(Δ/σ)_max_ = 0.009
311 parameters	Δρ_max_ = 1.83 e Å^−^^3^
19 restraints	Δρ_min_ = −0.86 e Å^−^^3^

### Special details

**Table:**

Geometry. All esds (except the esd in the dihedral angle between two l.s. planes) are estimated using the full covariance matrix. The cell esds are taken into account individually in the estimation of esds in distances, angles and torsion angles; correlations between esds in cell parameters are only used when they are defined by crystal symmetry. An approximate (isotropic) treatment of cell esds is used for estimating esds involving l.s. planes. Non-bonded distances: 6.1266 (0.0028) Au1 - O1 3.9827 (0.0004) Au1 - Au1_$2 3.6522 (0.0012) Au1 - Cl1_$2 Operator for generating equivalent atoms: $2 -x+1, -y+1, -z+1
Refinement. Refinement of F^2^ against ALL reflections. The weighted R-factor wR and goodness of fit S are based on F^2^, conventional R-factors R are based on F, with F set to zero for negative F^2^. The threshold expression of F^2^ > 2sigma(F^2^) is used only for calculating R-factors(gt) etc. and is not relevant to the choice of reflections for refinement. R-factors based on F^2^ are statistically about twice as large as those based on F, and R- factors based on ALL data will be even larger.

### Fractional atomic coordinates and isotropic or equivalent isotropic displacement parameters (Å^2^)

**Table:**

	*x*	*y*	*z*	*U*_iso_*/*U*_eq_	Occ. (<1)
Au1	0.52851 (2)	0.647310 (17)	0.545348 (14)	0.01888 (6)	
Cl1	0.27999 (14)	0.66076 (11)	0.48600 (9)	0.0244 (3)	
O1	0.4788 (3)	0.3560 (2)	0.9714 (2)	0.0145 (6)	
P1	0.51137 (12)	0.34200 (9)	0.86757 (8)	0.0095 (2)	
P2	0.72565 (12)	0.66749 (9)	0.73564 (8)	0.0098 (2)	
S1	0.78253 (13)	0.62695 (10)	0.60180 (8)	0.0180 (2)	
C1	0.6026 (5)	0.4594 (3)	0.7886 (3)	0.0107 (8)	
H1	0.6371	0.4502	0.7235	0.013*	
C2	0.6236 (5)	0.5582 (4)	0.8175 (3)	0.0119 (8)	
H2	0.5850	0.5714	0.8810	0.014*	
C11	0.3342 (5)	0.3399 (3)	0.8051 (3)	0.0099 (8)	
C12	0.2241 (5)	0.2717 (4)	0.8571 (3)	0.0170 (9)	
H12	0.2446	0.2293	0.9236	0.020*	
C13	0.0850 (5)	0.2652 (4)	0.8124 (3)	0.0195 (10)	
H13	0.0110	0.2186	0.8486	0.023*	
C14	0.0542 (5)	0.3263 (4)	0.7157 (3)	0.0175 (9)	
H14	−0.0405	0.3213	0.6851	0.021*	
C15	0.1620 (5)	0.3954 (4)	0.6629 (3)	0.0168 (9)	
H15	0.1398	0.4384	0.5967	0.020*	
C16	0.3017 (5)	0.4016 (4)	0.7066 (3)	0.0154 (9)	
H16	0.3756	0.4477	0.6698	0.018*	
C21	0.6553 (5)	0.2024 (3)	0.8576 (3)	0.0125 (8)	
C22	0.7163 (5)	0.1221 (4)	0.9446 (3)	0.0194 (10)	
H22	0.6842	0.1415	1.0072	0.023*	
C23	0.8246 (6)	0.0135 (4)	0.9384 (4)	0.0293 (12)	
H23	0.8643	−0.0428	0.9972	0.035*	
C24	0.8749 (5)	−0.0132 (4)	0.8477 (4)	0.0273 (12)	
H24	0.9498	−0.0873	0.8443	0.033*	
C25	0.8169 (5)	0.0676 (4)	0.7615 (4)	0.0251 (11)	
H25	0.8540	0.0498	0.6990	0.030*	
C26	0.7039 (5)	0.1753 (4)	0.7661 (3)	0.0162 (9)	
H26	0.6607	0.2293	0.7071	0.019*	
C31	0.9095 (5)	0.6644 (3)	0.7950 (3)	0.0113 (8)	
C32	1.0489 (5)	0.6829 (4)	0.7385 (4)	0.0195 (10)	
H32	1.0479	0.6954	0.6684	0.023*	
C33	1.1888 (5)	0.6829 (5)	0.7846 (4)	0.0253 (11)	
H33	1.2836	0.6963	0.7461	0.030*	
C34	1.1910 (5)	0.6638 (4)	0.8857 (4)	0.0196 (10)	
H34	1.2875	0.6637	0.9169	0.023*	
C35	1.0536 (5)	0.6445 (4)	0.9429 (3)	0.0199 (10)	
H35	1.0565	0.6307	1.0129	0.024*	
C36	0.9123 (5)	0.6454 (4)	0.8982 (3)	0.0168 (9)	
H36	0.8176	0.6332	0.9373	0.020*	
C41	0.5995 (5)	0.8183 (3)	0.7263 (3)	0.0110 (8)	
C42	0.5999 (6)	0.9051 (4)	0.6366 (3)	0.0192 (10)	
H42	0.6639	0.8825	0.5819	0.023*	
C43	0.5085 (6)	1.0225 (4)	0.6270 (3)	0.0212 (10)	
H43	0.5105	1.0808	0.5663	0.025*	
C44	0.4150 (5)	1.0543 (4)	0.7054 (4)	0.0197 (10)	
H44	0.3495	1.1343	0.6984	0.024*	
C45	0.4149 (6)	0.9712 (4)	0.7946 (4)	0.0286 (12)	
H45	0.3502	0.9949	0.8487	0.034*	
C46	0.5094 (5)	0.8522 (4)	0.8062 (3)	0.0212 (10)	
H46	0.5112	0.7958	0.8682	0.025*	
C99	0.0028 (15)	0.9195 (12)	0.5150 (9)	0.054 (3)*	0.50
H99A	−0.0639	0.8733	0.5649	0.065*	0.50
H99B	0.0642	0.8629	0.4745	0.065*	0.50
Cl98	0.1379 (12)	0.9738 (9)	0.5746 (7)	0.078 (3)	0.50
Cl99	−0.1245 (10)	1.0425 (5)	0.4385 (7)	0.062 (2)	0.50

### Atomic displacement parameters (Å^2^)

**Table:**

	*U*^11^	*U*^22^	*U*^33^	*U*^12^	*U*^13^	*U*^23^
Au1	0.02164 (10)	0.01867 (10)	0.01674 (10)	−0.00635 (7)	−0.00190 (6)	−0.00213 (6)
Cl1	0.0235 (6)	0.0274 (6)	0.0211 (6)	−0.0072 (5)	−0.0064 (5)	0.0010 (5)
O1	0.0136 (15)	0.0147 (15)	0.0163 (17)	−0.0042 (12)	−0.0004 (13)	−0.0047 (13)
P1	0.0093 (5)	0.0079 (5)	0.0118 (5)	−0.0035 (4)	0.0000 (4)	−0.0013 (4)
P2	0.0080 (5)	0.0087 (5)	0.0131 (5)	−0.0032 (4)	0.0001 (4)	−0.0022 (4)
S1	0.0168 (6)	0.0182 (6)	0.0192 (6)	−0.0034 (4)	0.0001 (5)	−0.0048 (5)
C1	0.0068 (19)	0.0104 (19)	0.014 (2)	−0.0013 (15)	−0.0019 (16)	−0.0005 (16)
C2	0.0043 (18)	0.012 (2)	0.019 (2)	−0.0013 (15)	0.0009 (16)	−0.0016 (17)
C11	0.0084 (19)	0.0082 (19)	0.014 (2)	−0.0006 (15)	0.0002 (16)	−0.0062 (16)
C12	0.021 (2)	0.017 (2)	0.014 (2)	−0.0090 (18)	−0.0002 (18)	−0.0010 (18)
C13	0.011 (2)	0.024 (2)	0.025 (3)	−0.0104 (18)	0.0016 (19)	−0.004 (2)
C14	0.009 (2)	0.018 (2)	0.026 (3)	0.0004 (17)	−0.0038 (18)	−0.0059 (19)
C15	0.017 (2)	0.016 (2)	0.016 (2)	−0.0004 (17)	−0.0038 (18)	0.0002 (18)
C16	0.013 (2)	0.015 (2)	0.018 (2)	−0.0067 (17)	0.0006 (17)	0.0012 (17)
C21	0.0093 (19)	0.0073 (19)	0.021 (2)	−0.0034 (15)	0.0005 (17)	−0.0024 (17)
C22	0.019 (2)	0.018 (2)	0.019 (2)	−0.0055 (18)	−0.0030 (19)	0.0028 (18)
C23	0.019 (2)	0.016 (2)	0.046 (3)	−0.0037 (19)	−0.003 (2)	0.007 (2)
C24	0.013 (2)	0.012 (2)	0.059 (4)	−0.0010 (18)	−0.002 (2)	−0.012 (2)
C25	0.013 (2)	0.027 (3)	0.042 (3)	−0.0050 (19)	0.003 (2)	−0.021 (2)
C26	0.013 (2)	0.016 (2)	0.022 (2)	−0.0047 (17)	−0.0028 (18)	−0.0066 (18)
C31	0.009 (2)	0.0075 (19)	0.017 (2)	−0.0012 (15)	−0.0030 (16)	−0.0033 (16)
C32	0.016 (2)	0.024 (2)	0.021 (2)	−0.0077 (19)	0.0019 (19)	−0.008 (2)
C33	0.010 (2)	0.038 (3)	0.032 (3)	−0.008 (2)	0.005 (2)	−0.015 (2)
C34	0.009 (2)	0.017 (2)	0.034 (3)	0.0005 (17)	−0.0092 (19)	−0.007 (2)
C35	0.023 (2)	0.017 (2)	0.019 (2)	−0.0057 (19)	−0.0070 (19)	0.0001 (19)
C36	0.016 (2)	0.016 (2)	0.018 (2)	−0.0066 (17)	−0.0020 (18)	0.0007 (18)
C41	0.011 (2)	0.0050 (18)	0.018 (2)	−0.0021 (15)	−0.0047 (17)	−0.0031 (16)
C42	0.025 (2)	0.021 (2)	0.010 (2)	0.0007 (19)	−0.0005 (18)	−0.0054 (18)
C43	0.031 (3)	0.009 (2)	0.020 (2)	0.0035 (19)	−0.012 (2)	0.0016 (18)
C44	0.016 (2)	0.010 (2)	0.031 (3)	0.0032 (17)	−0.003 (2)	−0.0059 (19)
C45	0.031 (3)	0.019 (2)	0.032 (3)	−0.002 (2)	0.019 (2)	−0.007 (2)
C46	0.024 (2)	0.015 (2)	0.021 (2)	−0.0052 (19)	0.008 (2)	0.0031 (18)
Cl98	0.065 (4)	0.134 (6)	0.061 (3)	−0.061 (4)	0.032 (2)	−0.046 (4)
Cl99	0.069 (4)	0.0233 (17)	0.082 (5)	−0.004 (2)	0.029 (3)	−0.004 (2)

### Geometric parameters (Å, º)

**Table:**

Au1—Cl1	2.2726 (12)	C24—H24	0.9500
Au1—S1	2.2846 (11)	C25—C26	1.396 (6)
O1—P1	1.484 (3)	C25—H25	0.9500
P1—C11	1.791 (4)	C26—H26	0.9500
P1—C1	1.803 (4)	C31—C32	1.394 (6)
P1—C21	1.811 (4)	C31—C36	1.399 (6)
P2—C31	1.801 (4)	C32—C33	1.385 (6)
P2—C41	1.803 (4)	C32—H32	0.9500
P2—C2	1.809 (4)	C33—C34	1.371 (7)
P2—S1	2.0135 (16)	C33—H33	0.9500
C1—C2	1.330 (6)	C34—C35	1.386 (6)
C1—H1	0.9500	C34—H34	0.9500
C2—H2	0.9500	C35—C36	1.382 (6)
C11—C12	1.397 (6)	C35—H35	0.9500
C11—C16	1.406 (6)	C36—H36	0.9500
C12—C13	1.391 (6)	C41—C46	1.379 (6)
C12—H12	0.9500	C41—C42	1.406 (6)
C13—C14	1.380 (6)	C42—C43	1.379 (6)
C13—H13	0.9500	C42—H42	0.9500
C14—C15	1.392 (6)	C43—C44	1.367 (6)
C14—H14	0.9500	C43—H43	0.9500
C15—C16	1.387 (6)	C44—C45	1.379 (7)
C15—H15	0.9500	C44—H44	0.9500
C16—H16	0.9500	C45—C46	1.402 (6)
C21—C26	1.387 (6)	C45—H45	0.9500
C21—C22	1.397 (6)	C46—H46	0.9500
C22—C23	1.391 (6)	C99—Cl98	1.747 (12)
C22—H22	0.9500	C99—Cl99	1.760 (11)
C23—C24	1.376 (8)	C99—H99A	0.9900
C23—H23	0.9500	C99—H99B	0.9900
C24—C25	1.384 (7)		
			
Cl1—Au1—S1	177.55 (4)	C25—C24—H24	119.9
O1—P1—C11	113.74 (17)	C24—C25—C26	120.2 (5)
O1—P1—C1	114.90 (18)	C24—C25—H25	119.9
C11—P1—C1	105.43 (19)	C26—C25—H25	119.9
O1—P1—C21	112.88 (18)	C21—C26—C25	119.3 (4)
C11—P1—C21	106.13 (18)	C21—C26—H26	120.3
C1—P1—C21	102.73 (18)	C25—C26—H26	120.3
C31—P2—C41	107.84 (18)	C32—C31—C36	119.7 (4)
C31—P2—C2	106.69 (19)	C32—C31—P2	120.2 (3)
C41—P2—C2	108.17 (18)	C36—C31—P2	120.1 (3)
C31—P2—S1	109.00 (14)	C33—C32—C31	119.9 (4)
C41—P2—S1	111.91 (15)	C33—C32—H32	120.1
C2—P2—S1	112.98 (15)	C31—C32—H32	120.1
P2—S1—Au1	100.06 (5)	C34—C33—C32	120.2 (4)
C2—C1—P1	122.9 (3)	C34—C33—H33	119.9
C2—C1—H1	118.5	C32—C33—H33	119.9
P1—C1—H1	118.5	C33—C34—C35	120.5 (4)
C1—C2—P2	120.0 (3)	C33—C34—H34	119.7
C1—C2—H2	120.0	C35—C34—H34	119.7
P2—C2—H2	120.0	C36—C35—C34	120.2 (4)
C12—C11—C16	118.6 (4)	C36—C35—H35	119.9
C12—C11—P1	117.9 (3)	C34—C35—H35	119.9
C16—C11—P1	123.5 (3)	C35—C36—C31	119.6 (4)
C13—C12—C11	120.8 (4)	C35—C36—H36	120.2
C13—C12—H12	119.6	C31—C36—H36	120.2
C11—C12—H12	119.6	C46—C41—C42	119.4 (4)
C14—C13—C12	120.1 (4)	C46—C41—P2	121.7 (3)
C14—C13—H13	119.9	C42—C41—P2	118.9 (3)
C12—C13—H13	119.9	C43—C42—C41	120.9 (4)
C13—C14—C15	119.9 (4)	C43—C42—H42	119.6
C13—C14—H14	120.0	C41—C42—H42	119.6
C15—C14—H14	120.0	C44—C43—C42	119.5 (4)
C16—C15—C14	120.3 (4)	C44—C43—H43	120.3
C16—C15—H15	119.9	C42—C43—H43	120.3
C14—C15—H15	119.9	C43—C44—C45	120.6 (4)
C15—C16—C11	120.3 (4)	C43—C44—H44	119.7
C15—C16—H16	119.9	C45—C44—H44	119.7
C11—C16—H16	119.9	C44—C45—C46	120.7 (4)
C26—C21—C22	120.5 (4)	C44—C45—H45	119.7
C26—C21—P1	121.1 (3)	C46—C45—H45	119.7
C22—C21—P1	118.4 (3)	C41—C46—C45	119.0 (4)
C23—C22—C21	119.1 (4)	C41—C46—H46	120.5
C23—C22—H22	120.5	C45—C46—H46	120.5
C21—C22—H22	120.5	Cl98—C99—Cl99	110.3 (9)
C24—C23—C22	120.6 (5)	Cl98—C99—H99A	109.6
C24—C23—H23	119.7	Cl99—C99—H99A	109.6
C22—C23—H23	119.7	Cl98—C99—H99B	109.6
C23—C24—C25	120.2 (4)	Cl99—C99—H99B	109.6
C23—C24—H24	119.9	H99A—C99—H99B	108.1
			
C31—P2—S1—Au1	−177.51 (14)	C22—C23—C24—C25	−0.6 (7)
C41—P2—S1—Au1	−58.32 (15)	C23—C24—C25—C26	−1.6 (7)
C2—P2—S1—Au1	64.07 (15)	C22—C21—C26—C25	−1.1 (6)
Cl1—Au1—S1—P2	−143.9 (10)	P1—C21—C26—C25	179.1 (3)
O1—P1—C1—C2	−6.9 (4)	C24—C25—C26—C21	2.4 (6)
C11—P1—C1—C2	119.2 (4)	C41—P2—C31—C32	−97.6 (4)
C21—P1—C1—C2	−129.9 (4)	C2—P2—C31—C32	146.4 (3)
P1—C1—C2—P2	176.8 (2)	S1—P2—C31—C32	24.1 (4)
C31—P2—C2—C1	−115.7 (3)	C41—P2—C31—C36	81.3 (4)
C41—P2—C2—C1	128.5 (3)	C2—P2—C31—C36	−34.7 (4)
S1—P2—C2—C1	4.1 (4)	S1—P2—C31—C36	−157.0 (3)
O1—P1—C11—C12	−42.7 (4)	C36—C31—C32—C33	−0.3 (6)
C1—P1—C11—C12	−169.4 (3)	P2—C31—C32—C33	178.6 (3)
C21—P1—C11—C12	82.0 (3)	C31—C32—C33—C34	0.6 (7)
O1—P1—C11—C16	138.3 (3)	C32—C33—C34—C35	−0.2 (7)
C1—P1—C11—C16	11.5 (4)	C33—C34—C35—C36	−0.5 (7)
C21—P1—C11—C16	−97.0 (4)	C34—C35—C36—C31	0.7 (6)
C16—C11—C12—C13	−0.2 (6)	C32—C31—C36—C35	−0.4 (6)
P1—C11—C12—C13	−179.3 (3)	P2—C31—C36—C35	−179.3 (3)
C11—C12—C13—C14	0.1 (7)	C31—P2—C41—C46	−78.6 (4)
C12—C13—C14—C15	−0.5 (7)	C2—P2—C41—C46	36.4 (4)
C13—C14—C15—C16	1.0 (6)	S1—P2—C41—C46	161.5 (3)
C14—C15—C16—C11	−1.1 (6)	C31—P2—C41—C42	98.0 (4)
C12—C11—C16—C15	0.7 (6)	C2—P2—C41—C42	−146.9 (3)
P1—C11—C16—C15	179.7 (3)	S1—P2—C41—C42	−21.8 (4)
O1—P1—C21—C26	−178.3 (3)	C46—C41—C42—C43	−1.5 (7)
C11—P1—C21—C26	56.5 (4)	P2—C41—C42—C43	−178.3 (4)
C1—P1—C21—C26	−54.0 (4)	C41—C42—C43—C44	−0.7 (7)
O1—P1—C21—C22	1.9 (4)	C42—C43—C44—C45	1.8 (7)
C11—P1—C21—C22	−123.4 (3)	C43—C44—C45—C46	−0.7 (8)
C1—P1—C21—C22	126.2 (3)	C42—C41—C46—C45	2.7 (7)
C26—C21—C22—C23	−1.0 (6)	P2—C41—C46—C45	179.3 (4)
P1—C21—C22—C23	178.9 (3)	C44—C45—C46—C41	−1.6 (8)
C21—C22—C23—C24	1.8 (7)		

### Hydrogen-bond geometry (Å, º)

**Table:**

*D*—H···*A*	*D*—H	H···*A*	*D*···*A*	*D*—H···*A*
C2—H2···O1^i^	0.95	2.36	3.294 (5)	166
C46—H46···O1^i^	0.95	2.49	3.438 (5)	179
C26—H26···Cl1^ii^	0.95	2.75	3.583 (5)	147
C34—H34···O1^iii^	0.95	2.54	3.478 (5)	170

Symmetry codes: (i) −*x*+1, −*y*+1, −*z*+2; (ii) −*x*+1, −*y*+1, −*z*+1; (iii) −*x*+2, −*y*+1, −*z*+2.

## References

[bb1] Agilent (2010). *CrysAlis PRO*. Agilent Technologies Ltd, Yarnton, Oxfordshire, England.

[bb2] Groom, C. R. & Allen, F. H. (2014). *Angew. Chem. Int. Ed.* **53**, 662–671. 10.1002/anie.201306438 24382699

[bb3] Groom, C. R., Bruno, I. J., Lightfoot, M. P. & Ward, S. C. (2016). *Acta Cryst.* B**72**, 171–179. 10.1107/S2052520616003954 PMC4822653 27048719

[bb4] Sheldrick, G. M. (2008). *Acta Cryst.* A**64**, 112–122. 10.1107/S0108767307043930 18156677

[bb5] Siemens (1994). *XP*. Siemens Analytical X-ray Instruments Inc., Madison, Wisconsin, USA.

[bb6] Sithole, S. V., Staples, R. J. & van Zyl, W. E. (2016). *Inorg. Chem. Commun.* **15**, 216–220.

[bb7] Taouss, C. & Jones, P. G. (2014). *Z. Naturforsch. Teil B*, **69**, 25–48.

[bb8] Taouss, C. & Jones, P. G. (2016). *Z. Naturforsch. Teil B*, **71**, 249–265.

